# Drug-Induced Lupus Erythematosus Associated with Antiretroviral Therapy in a Patient with Human Immunodeficiency Virus: A Case Report

**DOI:** 10.7759/cureus.1661

**Published:** 2017-09-07

**Authors:** Jazila Mantis, Ravi Bhavsar, Adriana Abrudescu

**Affiliations:** 1 Infectious Disease, Mount Sinai Icahn School of Medicine Queens Hospital New York; 2 Internal Medicine, Mount Sinai Icahn School of Medicine Queens Hospital New York; 3 Rheumatology, Mount Sinai Icahn School of Medicine Queens Hospital New York

**Keywords:** drug induced lupus erythematosus, antiretroviral therapy, hiv treatment, human immunodeficiency virus (hiv)

## Abstract

Antiretroviral medications are the mainstay of human immunodeficiency virus (HIV) therapy and some have been in use for over 20 years. To date, there have been no reported cases of antiretroviral therapy (ART) induced drug-induced lupus erythematosus (DILE). We present a case of a 35-year-old woman who received a combination of emtricitabine, rilpivirine, and tenofovir disoproxil fumarate for HIV treatment. Three years later, she developed an extensive rash and polyarthralgia in her extremities with laboratory findings significant for positive antinuclear antibody (ANA), anti-double stranded deoxyribonucleic acid (DNA) antibody (anti-dsDNA), and anti-histone antibody titers. Her systemic symptoms and rash improved with ART discontinuation. She was later restarted on her original ART due to difficulty in tolerating a different combination therapy. A few months after restarting ART, she developed new dermatologic symptoms, worsening arthralgias, tenderness of the metacarpophalangeal and proximal interphalangeal joints of the hand, and an increase in anti-dsDNA titers to 286 IU/ml. ART was then discontinued, which led to complete resolution of her symptoms and her anti-dsDNA decreased significantly. She had no further recurrence of symptoms. Awareness of the possibility of ART-induced DILE in the right clinical setting would prompt early recognition and management of this condition.

## Introduction

Drug-induced lupus erythematosus (DILE) is a rare syndrome occurring as an adverse reaction to certain medications, whereby autoantibodies are formed after continuous drug exposure leading to an autoimmune disorder similar to idiopathic lupus erythematosus (LE). DILE was first recognized in 1945 with sulfadiazine as the offending agent [1]. Since then, more than 90 medications from more than 10 drug classes have been implicated in causing DILE [2]. It is estimated that approximately over 10% of LE cases are drug-induced [1]. Here, we report a rare case of DILE due to antiretroviral therapy (ART) in a patient with human immunodeficiency virus (HIV). To our knowledge, no such cases have yet been reported in the literature.

## Case presentation

A 35-year-old Guyanese woman was diagnosed with HIV infection nine years earlier, during pregnancy, as part of her prenatal care with a CD4 count of 716 (37%) and a viral load of 51 copies/ml. She received ART with a combination of lamivudine/zidovudine, ritonavir, and saquinavir for four months, which was stopped after delivery. ART was then resumed after six years with emtricitabine, rilpivirine, and tenofovir disoproxil fumarate when her CD4 count trended down to 482 (31%). She had no other medical problems and was on no other medication.

Three years after starting ART, she developed a hyperpigmented erythematous macular rash on her forearm along with hypopigmented atrophic patches bilaterally on the anterior segment of her shins. She also had extensive hair loss, polyarthralgia involving the shoulder, elbow, and wrist joints, along with exertional shortness of breath and weight loss. Her HIV disease was stable with an undetectable viral load and a CD4 count of 591 (44%). Laboratory findings were significant for a positive antinuclear antibody (ANA) titer of 1:320 (homogeneous pattern), a positive anti-double-stranded DNA antibody (anti-dsDNA) of EIA 103 IU/mL, and an anti-histone antibody reported as strongly positive at 5.3 units. Anti-Smith, anti-ribonucleoprotein (RNP), and anti-Sjögren syndrome A/B antibodies were negative. Her complement levels remained normal throughout the course of her illness. A skin biopsy of the rash showed vacuolar interface alteration as well as superficial and deep perivascular and perifollicular lymphocytic infiltrate. A computed tomography (CT) scan of the chest, abdomen, and pelvis showed diffuse mediastinal, supraclavicular, and inguinal lymphadenopathy. A biopsy of an inguinal lymph node showed reactive lymphoid hyperplasia. Urine analysis and renal functions were normal. The patient was started on hydroxychloroquine 200 mg orally twice daily followed by discontinuation of ART. There was a significant improvement in her systemic symptoms and her rash.

She was later given a combination of dolutegravir, abacavir, and lamivudine to which she developed severe pruritus within a few days, leading to the discontinuation of the regimen. The patient subsequently requested a trial of her former regimen of emtricitabine, rilpivirine, and tenofovir disoproxil fumarate, which was restarted.

After a few months of restarting emtricitabine, rilpivirine, and tenofovir disoproxil fumarate, the patient developed a hyperpigmented plaque on the vertex of her skull measuring 1.6 cm x 1.4 cm and a similar lesion on the posterior region of her right leg, with worsening arthralgias in her shoulders, wrists, and elbow joints. Physical examination revealed tenderness of the metacarpophalangeal and proximal interphalangeal joints of the hand. Anti-dsDNA titer was measured at 286 IU/ml and anti-histone antibodies were positive at 2.5 units. Additionally, she also complained of blurry vision in her left eye and was referred to ophthalmology. A magnetic resonance imaging (MRI) scan of the orbit revealed no intra/extra conal focal mass lesions or collections with no abnormal enhancements. Hydroxychloroquine was discontinued as a precautionary measure, and ART was discontinued in view of her symptoms and high lupus titers, which then led to complete resolution of her joint pains and rash.

Four months after the second discontinuation of ART, the patient had no recurrence of her symptoms, and her anti-dsDNA and anti-histone antibody titers decreased to 71 IU/ml and 1.7 units, respectively. Her clinical findings and the complete remission of symptoms merited the diagnosis of DILE. The patient is currently off her ART medications and hydroxychloroquine and is receiving Prednisone 10 mg for the time being.

## Discussion

DILE is defined as a lupus-like syndrome occurring as an adverse reaction temporally related to continuous drug exposure, which resolves after the discontinuation of the drug. Generally, the symptoms related to the offending agent resolve after discontinuation of the drug. Although the exact pathogenesis responsible for DILE remains unclear, genetic predisposition is known to have a role.

The presentation of DILE can be extremely varied depending upon the type, frequency, and duration of the offending drug. DILE can present either as systemic drug-induced lupus or as subacute/chronic cutaneous lupus. The systemic form commonly presents with fever, myalgias, rash, arthralgias, and serositis and rarely with severe involvement of renal, nervous and gastrointestinal systems [1,3]. Drugs that have been studied to have the highest risk of systemic DILE are procainamide, hydralazine, penicillamine, isoniazid, quinidine, anti-tumor necrosis factor inhibitors, interferon alpha, methyldopa, diltiazem, and chlorpromazine [4]. 

On the other hand, the subacute cutaneous form (SCLE) is the most common variant of DILE presenting with symmetric, annular papulosquamous lesions, usually in sun-exposed areas of the body, with a strong predilection for lower extremities [4]. This feature is not commonly seen in idiopathic lupus. In our case, the patient exhibited skin lesions on her legs when she was exposed to antiretroviral medications. Although SCLE has been associated with thiazide diuretics, calcium channel blockers, and angiotensin-converting enzyme inhibitors, there have been no cases reported with HIV therapy [3]. The mechanisms by which this variant of DILE exhibits itself largely continues to be unclear. Hypotheses have been made regarding immune responses to self-antigens and the nonimmune cytotoxicity caused by offending agents being responsible for the skin lesions [3].

In DILE, ANA has been invariably found to be homogeneous, in contrast with the ANA pattern in idiopathic LE, which can be homogeneous or speckled [1]. Anti-histone antibodies are positive in 95% of the cases, and ANA antibodies are usually positive with negative anti-Smith and generally low or absent anti-double stranded DNA antibodies, with the exception of DILE induced by TNF-ɑ inhibitors for which anti-dsDNA antibodies may be present in 90% of the cases [4]. Our patient had high anti-dsDNA titers temporally related to her exposure to ART similar to high anti-dsDNA antibodies seen in DILE associated with tumor necrosis factor alpha (TNF-ɑ) inhibitors.

There are no definitive diagnostic criteria for DILE, but the following guidelines have been proposed: (a) sufficient and continuing exposure to a specific drug, (b) at least one symptom compatible with systemic lupus erythematous (SLE), (c) no history suggestive of SLE before starting the drug, and (d) resolution of symptoms within weeks (sometimes months) after discontinuation of the offending agent [5]. Our patient had no features suggestive of lupus before the initiation of ART and all of her symptoms completely resolved on discontinuation of the offending agents.

## Conclusions

Antiretroviral medications are the mainstay of HIV treatment and, to our knowledge, there have been no cases of antiretroviral medication-associated DILE reported in the literature. Awareness of the possibility of ART-induced DILE in the right clinical setting would prompt early recognition and management of this condition.
